# Supplementary material for: Cohort profile: The Clinical and Multi-omic (CAMO) cohort, part of the Norwegian Women and Cancer (NOWAC) study
Source: PLoS One. 2023 Feb 6;18(2):e0281218. doi: 10.1371/journal.pone.0281218 (PMC9901780; doi:10.1371/journal.pone.0281218)
Supplement: S1 Appendix — (DOCX) [file pone.0281218.s001.docx]

# **S1 Appendix. Analyses of blood samples and tumor tissue.**

## **Analyses of blood samples**

From the 388 women in the clinical cohort, a set blood samples were collected as part of the NOWAC Post-genome cohort, see main text for details on the sample collection procedure.

For mRNA gene expression analyses, total RNA was isolated in accordance with the manufacturer’s protocol (PAXgene Blood miRNA isolation Kit, Preanalytix/Qiagen, Hilden, Germany). The mRNA was amplified and labelled using the Illumina TotalPrepT-96 RNA Amplification Kit (Ambion Inc., Austin, TX, USA), and hybridized to Illumina HumanHT-12 Expression BeadChip microarrays (Illumina, Inc. San Diego, CA, USA). Lab work was carried out at a certified Illumina service provider, the Genomics Core Facility (GCF), Norwegian University of Science and Technology (NTNU), Trondheim, Norway.

In plasma samples, both microRNA and metabolomics were analyzed. For metabolomics, the liquid chromatography-mass spectrometry (LC-MS/MS) based kit “AbsoluteIDQ p180” (Biocrates Life Sciences, Innsbruck, Austria)) was used for quantification of up to 188 metabolites. The Swedish Metabolomics Centre in Umeå, Sweden carried out the analyses.

For plasma microRNA profiling, the plasma was thawed and centrifuged at 3000 x g for 5 min, and 60 μl of Lysis solution BF containing 1μg carrier-RNA per 60μl Lysis Solution BF and RNA spike-in template mixture was added, followed by addition of 20 μL Protein Precipitation solution BF. Total RNA was extracted from the samples using miRCURY RNA isolation Kit – Biofluids; high-throughput bead-based protocol v.1 (Exiqon, Vedbaek, Denmark). A PCR-based panel of 372 probes (miRCURY LNATM Universal RT microRNA PCR Human panel I, Qiagen, Hilden, Germany) was used for microRNA profiling. Lab work was carried out by Exiqon AS, Denmark.

## **Analyses of tumor tissue**

FFPE and HE slides were retrieved from the pathology labs. Tumor grade, ER, PR, Her2 and Ki67 were analyzed as part of routine diagnostics, as described in the main text.

Tissue microarrays (TMAs) were constructed as described in the main text and used for high-throughput analysis of molecular markers using HE staining, IHC, and ISH. Molecular marker levels were scored according to the following general protocol: Viable tissue cores were scored manually and semi-quantitatively for staining density and/or intensity in a four-tiered ordinal scale (0-3). Three researchers carry out the scoring independently. All samples are anonymized. When assessing a given core, the observers are blinded to the scores of the other observers and to the outcome of the patient. Typically, each researcher scores the staining density, i.e. number or percentage of positive cells, and/or intensity, i.e. weak, moderate or strong staining, in each core. Further, since different tissue and cellular departments can be identified in each core, the scores can be given to stroma and tumor tissue separately, or even to tumor cell cytoplasm and nuclei separately. The scores are subsequently used to calculate the mean stroma, tumor cytoplasm and tumor nucleus score for each case. In case of disagreement (score discrepancy > 1), the slides are re-examined until a consensus is reached by the observers. Currently, scoring of the following markers is ongoing: miR-17-5p miR-20a-5p, CD4, CD8, CD34, CD66b, CD276/B7H3, TCRγδ and interleukin (IL)-17.

Total RNA from FFPE tissue blocks was extracted using the Recover All™ Total Nucleic Acid Isolation Kit for FFPE Tissues protocol (Ambion, Thermo Fisher, Loughborough, England). The samples were labeled using the miRCURY LNA™ microRNA Hi-Power Labeling Kit, Hy3™/Hy5™, and hybridized on the miRCURY LNA™ microRNA Array (7th Gen), which contained 3100 capture probes covering 94% of the human miRNAs in miRBASE version 19.0. RT-qPCR was performed on 40 tumor samples and 20 benign tissue controls to validate 15 selected miRNAs. This work was carried out by Exiqon AS, Denmark.
